# The CHA_2_DS_2_-VAS_C_ Score Predicts Mortality in Patients Undergoing Coronary Angiography

**DOI:** 10.3390/life13102026

**Published:** 2023-10-09

**Authors:** Nicholay Teodorovich, Gera Gandelman, Michael Jonas, Yakov Fabrikant, Michael Sraia Swissa, Sara Shimoni, Jacob George, Moshe Swissa

**Affiliations:** 1 Kaplan Medical Center, Rehovot and the Hebrew University, Jerusalem 7661041, Israel; gera_g@clalit.org.il (G.G.); michaelyo2@clalit.org.il (M.J.); yakovfa@clalit.org.il (Y.F.); ashimoni@zahav.net.il (S.S.); kobige@clalit.org.il (J.G.); swissam@clalit.org.il (M.S.); 2 Shari-Zedek Medical Center, and the Hebrew University, Jerusalem 9103102, Israel; sraia8@gmail.com

**Keywords:** CHA_2_DS_2_-VAS_C_ score, coronary angiography, outcomes, mortality

## Abstract

Background: The CHA_2_DS_2_-VAS_C_ score is used to predict the risk of thromboembolic complications in patients with atrial fibrillation (AF). We hypothesized that the CHA_2_DS_2_-VAS_C_ score can be used to predict mortality in patients undergoing coronary angiography. Methods and Results: This was a prospective study of 990 patients undergoing coronary angiography. The median follow-up was 2294 days. The patients were categorized into two groups according to their CHA_2_DS_2_-VAS_C_ score: group I had scores <4 and group II had scores ≥4 (527 (53.2%) and 463 (46.8%), respectively). A Kaplan–Meier analysis demonstrated a significant association between the CHA_2_DS_2_-VAS_C_ score and mortality (69/527 (13.1%) vs. 179/463 (38.7%) for group I vs. group II, respectively, *p* < 0.0001). The association remained significant in patients with and without AF, reduced and preserved LVEF, normal and reduced kidney function, and with and without ACS (*p* < 0.009 to *p* < 0.0001 for all). In the Cox regression model, which combined the CHA_2_DS_2_-VAS_C_ score, the presence of AF, LVEF, anemia, and renal insufficiency, an elevated CHA_2_DS_2_-VAS_C_ score of ≥4 was independently associated with higher mortality (HR 2.12, CI 1.29–3.25, *p* = 0.001). Conclusions: The CHA_2_DS_2_VAS_C_ score is a simple and reliable mortality predictor in patients undergoing coronary angiography and should be used for the initial screening for such patients.

## 1. Introduction

The CHA_2_DS_2_-VAS_C_ score was developed to predict the probability of stroke in patients with atrial fibrillation and guide their anticoagulation therapy [1,2,3]. It represents a refinement of the previously used CHADS_2_ score, providing superior risk stratification [2]. Recently, the use of CHA_2_DS_2_-VAS_C_ has expanded and was demonstrated to predict the development of atrial fibrillation [4,5], left atrial dysfunction [6], ablation outcomes [7], estimate stroke severity [8,9] and its mechanism [10], and predict the occurrence of stroke in patients without atrial fibrillation [11,12,13,14]. CHA_2_DS_2_-VAS_C_ also correlates with the presence of the coronary artery disease [15,16,17], pulmonary embolism [18], and mortality [14,19,20,21,22,23,24,25]. In patients with established coronary artery disease, the CHA_2_DS_2_-VAS_C_ score has been demonstrated to predict the development of atrial fibrillation [26,27,28], ischemic severity [29,30], and stroke [27,31,32,33,34]. CHA_2_DS_2_-VAS_C_ also been shown to estimate the prognosis and predict the mortality of these patients [34,35,36,37,38,39,40,41]. However, almost all studies were done on patients with acute coronary syndromes. There is a paucity of data considering the usefulness of the CHA_2_DS_2_-VAS_C_ score to predict mortality in patients with the coronary artery disease in a nonurgent setting.

We hypothesized that the CHA_2_DS_2_-VAS_C_ score can be a useful tool to predict mortality in patients undergoing coronary angiography in both urgent (i.e., ACS) and elective scenarios.

## 2. Materials and Methods

This is a single center prospective observational study of 990 consecutive patients who underwent coronary angiography at the Kaplan Medical Center, Rehovot, Israel. The data were collected from the patients and their medical records at the time of admission. Informed consent was obtained from all patients. The computerized records and the national population registry database were used in the follow-up. The data included age, sex, coronary risk factors, detailed history of coronary artery disease, presence of atrial fibrillation, presence of heart failure, previous stroke and PVD, laboratory and echocardiographic data, indication for coronary angiography, its result, and the details of the intervention, among others.

Baseline patient clinical characteristics and procedural data were compared between patients in the two groups, according to the CHA_2_DS_2_-VAS_C_ score. The chi-square test was used for dichotomous variables, and an independent *t*-test was used for continuous variables. Data are expressed as the mean ± SD or frequency and percentage when appropriate. The correlation of the ascending (0–9) values of the CHA_2_DS_2_-VAS_C_ score with mortality was determined with the chi-square test, and the optimal cutoff value of the CHA_2_DS_2_-VAS_C_ score for mortality prediction was done with c-statistics. Cumulative event proportions were calculated by the Kaplan–Meier method, and the outcome differences of the two CHA_2_DS_2_-VAS_C_ groups were assessed with the log-rank test. Cox regression was utilized to access the independent value of the CHA_2_DS_2_-VAS_C_ score for mortality prediction. The covariates included in the multivariate model were identified using candidate variables that were predictive of the endpoint in the univariate analysis and were unbalanced between the two groups. The individual components of the CHA_2_DS_2_-VAS_C_ score were omitted from the multivariate model. A *p*-value <0.05 was considered significant. After analysis of the whole patient population, a Kaplan–Meier survival analysis was used to assess the survival in the specific subgroups of patients according to the presence or absence of AF, CHF, ACS, or CKD. Data were analyzed using SPSS statistical software version 21 and Medcalc version 17.5.5.3.

## 3. Results

Nine hundred and ninety consecutive patients undergoing coronary angiography electively or for acute coronary syndromes were followed up for a median of 2294 days. The distribution of the CHA_2_DS_2_-VAS_C_ scores is shown in Table 1. The mean CHADSVASC score was 3.35 ± 1.71, and the median was 3.0. Due to the low number of patients with CHA_2_DS_2_-VAS_C_ scores of 8 and 9, we combined the patients with scores 7 and above into one group. Mortality increased with the increasing CHA_2_DS_2_-VAS_C_ scores up to 6, with a similar rate in patients with scores 7+.

After Bonferroni correction, a significant (*p* < 0.05) difference in mortality was observed between CHA_2_DS_2_-VAS_C_ scores in the 0–3, 4, and 5–7 groups. All individual components of the CHA_2_DS_2_-VAS_C_ score, apart from previous stroke, were significantly associated with mortality (Table 2). In the multivariate analysis, all individual components of the CHA_2_DS_2_-VAS_C_ score, besides gender and previous stroke, were significantly associated with mortality.

**Figure 1:**
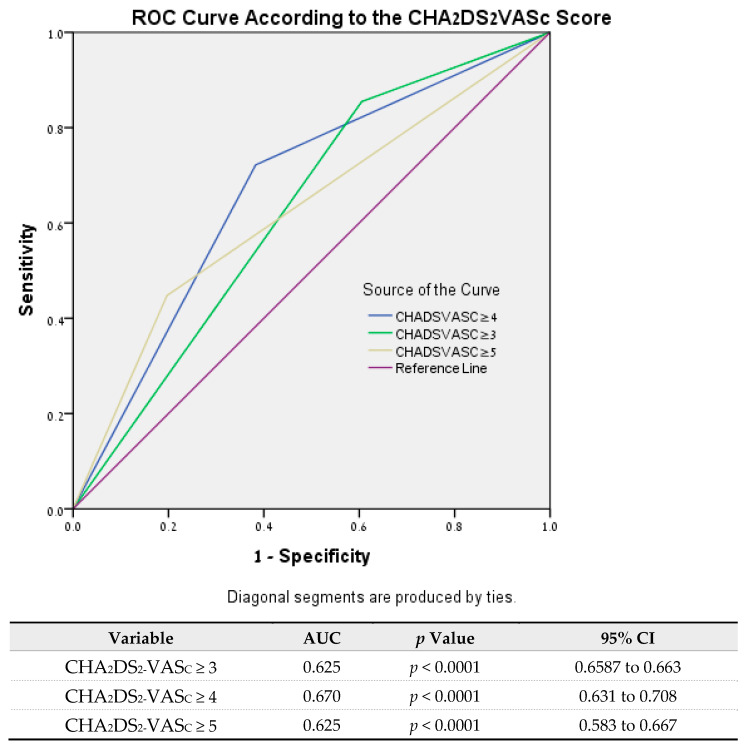
Predictive probability of mortality of CHA_2_DS_2_-VAS_C_ scores of 3, 4, and 5.

C statistics demonstrated the superiority of the CHA_2_DS_2_VAS_C_ score of 4 vs. 3 or 5 as the optimal cutoff for mortality prediction with an AUC = 0.67 (shown in Figure 1).

Based on that data, we divided the patients into two groups, according to their CHA_2_DS_2_-VAS_C_ scores (<4 and equal or ≥4). Patients with CHA_2_DS_2_-VAS_C_ scores < 4 were younger, had a lower incidence of prior coronary or vascular disease, and better renal function. They were less likely to have atrial fibrillation, obstructive CAD, and calcified lesions on their coronary angiography. The two groups were not significantly different in left ventricular function and acute coronary syndrome (ACS) as an indication for a coronary angiogram. The patients with CHA_2_DS_2_-VAS_C_ ≥ 4 had three-fold higher mortality than the patients in group I. The details of the differences between the groups are shown in Table 3.

A univariate analysis demonstrated a significant association of the elevated CHA_2_DS_2_-VAS_C_ score with mortality. Increased mortality was also associated with elevated age, the presence of hypertension, diabetes, heart failure, peripheral vascular disease, atrial fibrillation, renal failure, coronary artery calcification, decreased ejection fraction, significant diastolic dysfunction (elevated filling pressure), aortic stenosis, and anemia. Of note, the history of the myocardial infarction, previous PCI or CABG, acute coronary syndrome as a reason for coronary angiography, and the presence of obstructive coronary artery disease were not associated with increased mortality. A detailed description of the association of different demographic, clinical, laboratory, and angiographic variables with mortality is shown in Table 4.

**Figure 2:**
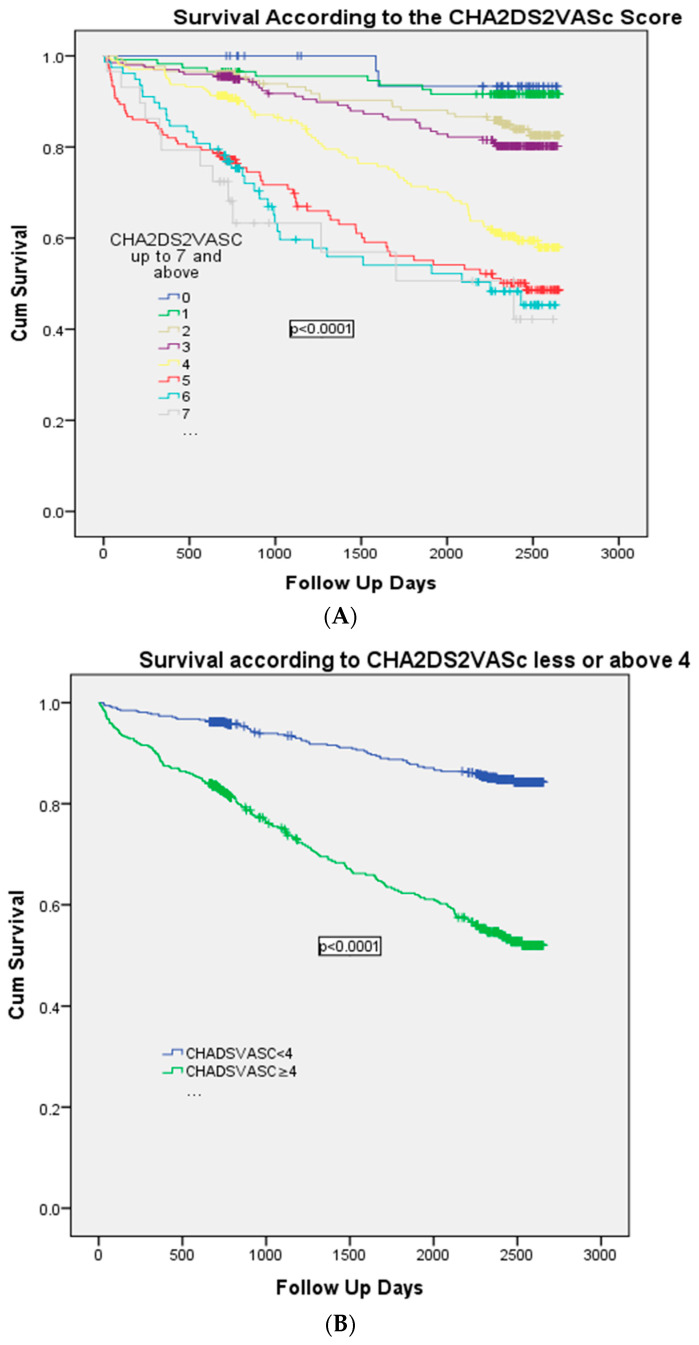
(**A**) Kaplan–Meier survival analysis according to the CHA_2_DS_2_-VAS_C_ score. (**B**) Kaplan–Meier survival analysis according to CHA_2_DS_2_-VAS_C_ scores < or ≥4.

The Kaplan–Meier analysis demonstrated a significant association (*p* < 0.0001) between the CHA_2_DS_2_-VAS_C_ score and mortality in general and when divided as CHA_2_DS_2_-VAS_C_ scores <4 vs. CHA_2_DS_2_-VAS_C_ scores ≥4: 69/527 (13.1%) vs. 179/463 (38.7%), respectively, *p* < 0.0001, as shown in Figure 2A,B).

In the Cox regression model, which combined the CHA_2_DS_2_-VAS_C_ score, presence of AF, LVEF, anemia, presence of aortic stenosis, and decreased GFR (<60 mL/min), an elevated CHA_2_DS_2_-VAS_C_ score of ≥4 was independently associated with higher mortality (hazard ratio 2.14, CI 1.40–3.256, *p* = <0.0001, as shown in Table 5).

### Subgroup Analysis

**Figure 3:**
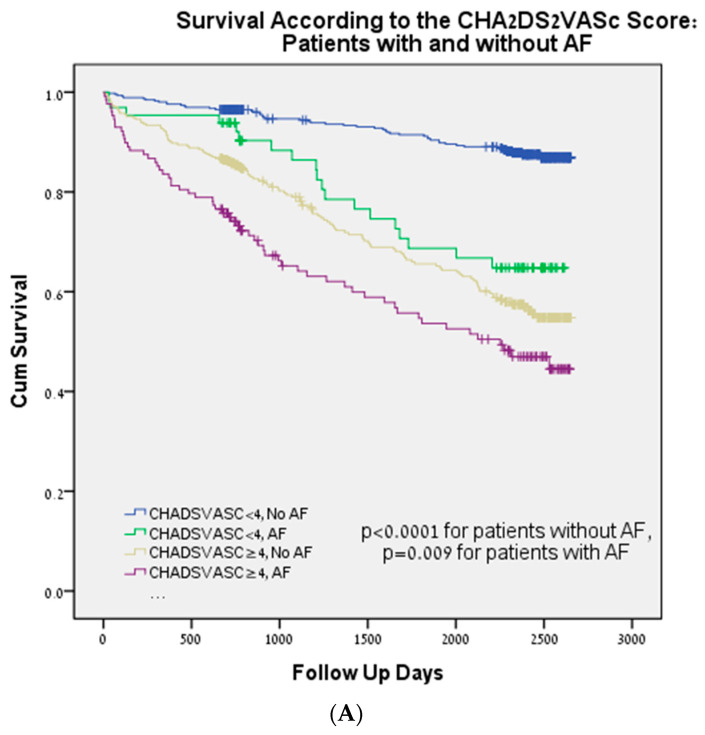

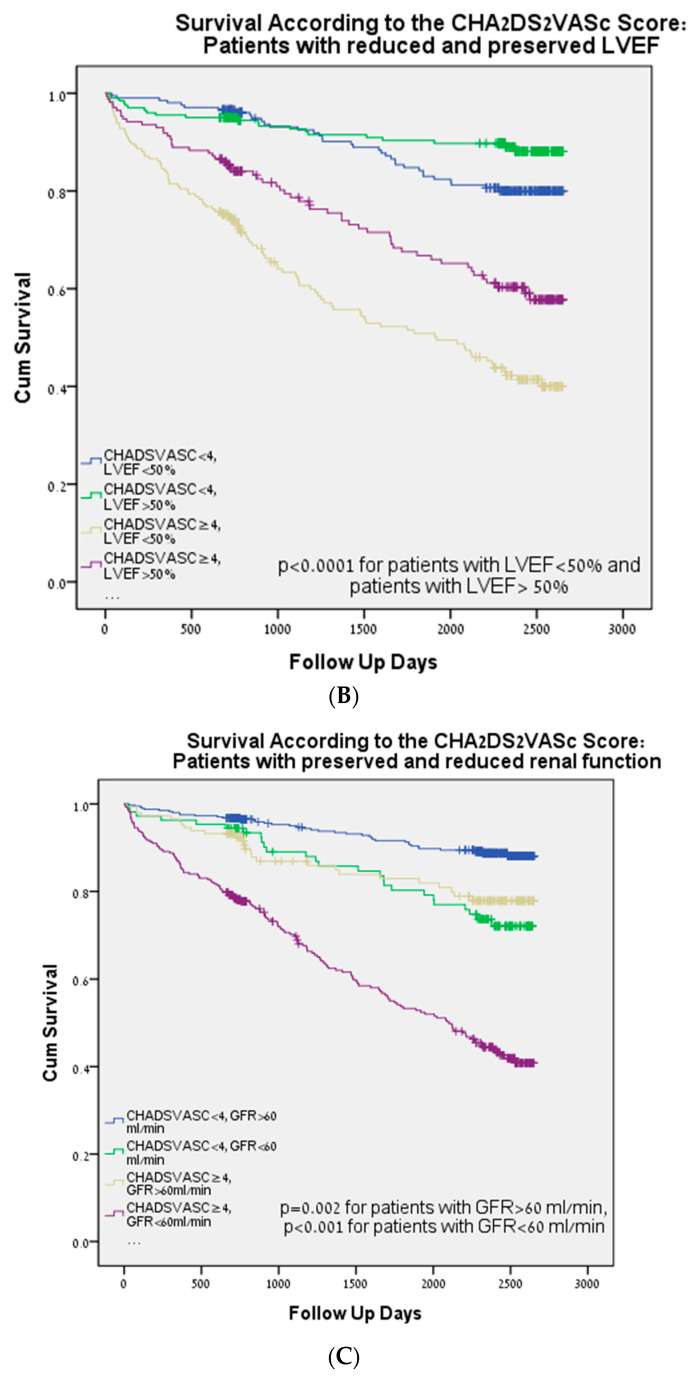

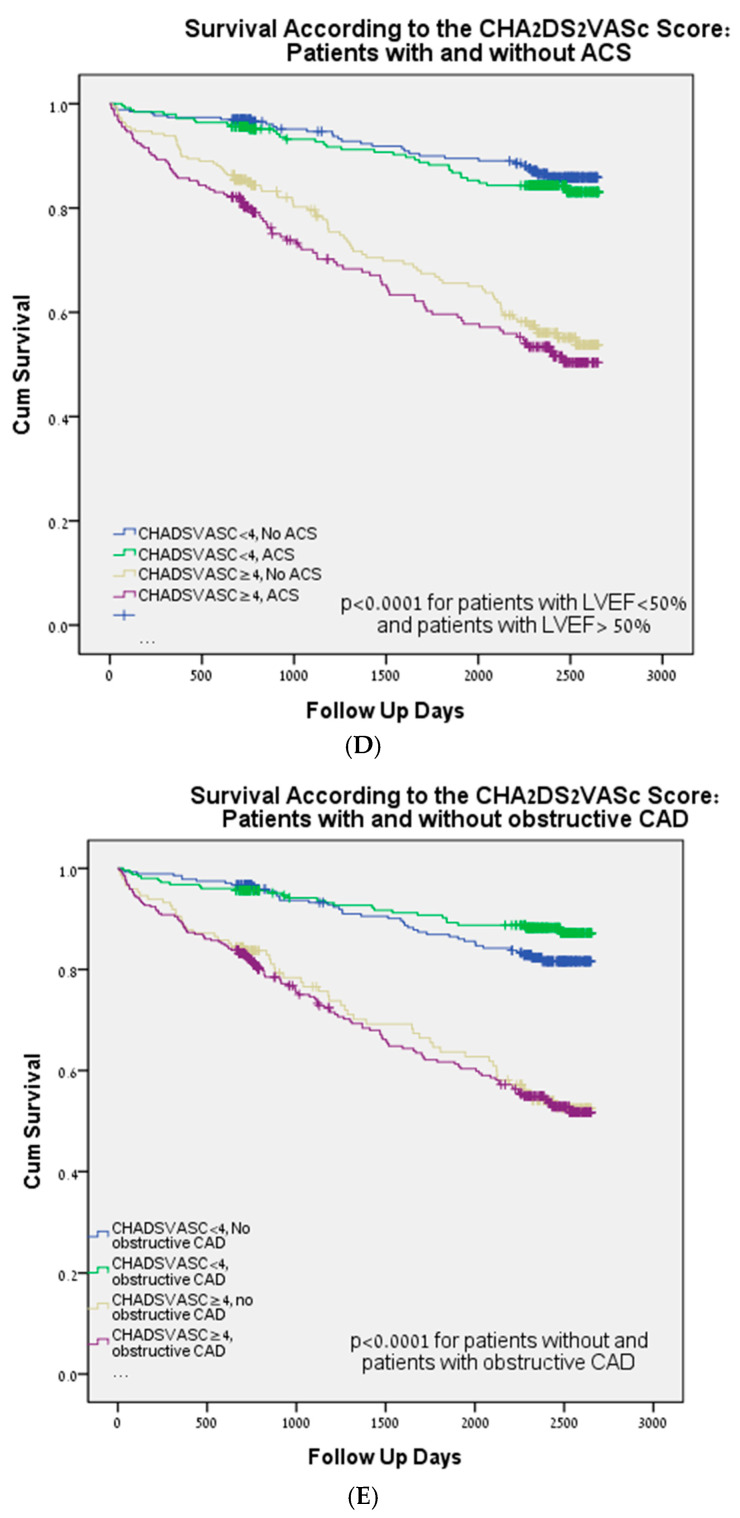
Kaplan–Meier survival analysis according to CHA_2_DS_2_-VAS_C_ scores < 4 or ≥4. (**A**) Patients with and without AF. (**B**) Patients with preserved and reduced LVEF. (**C**) Patients with normal and reduced kidney function. (**D**) Patients with and without acute coronary syndrome. (**E**) Patients with and without obstructive CAD on their coronary angiography.

We applied the Kaplan–Meier survival analysis to the different subgroups of the initial study cohort. The association between the CHA_2_DS_2_-VAS_C_ score (<4 vs. ≥4) and mortality remained significant in patients with and without AF (*p* < 0.009 and *p* < 0.0001, respectively), as shown in Figure 3A, with reduced and preserved LVEF (*p* < 0.0001 and *p* = 0.001, respectively), as shown in Figure 3B, normal and reduced GFR (*p* = 0.002 and *p* < 0.0001, respectively), as shown in Figure 3C, with and without ACS (*p* < 0.0001 for both groups), as shown in Figure 3D, and with nonobstructive and obstructive CAD on their coronary angiography (*p* < 0.0001 for both groups), as shown in Figure 3E.

Also, when the previously described Cox regression model (CHA_2_DS_2_-VAS_C_ score, presence of AF, LVEF, aortic stenosis, anemia, and presence of reduced GFR) was applied to the same groups, elevated CHA_2_DS_2_VAS_C_ scores of ≥4 were independently associated with higher mortality in most of the defined subgroups (shown in Table 6).

To further examine the usefulness of the CHA_2_DS_2_-VAS_C_ score for mortality prediction in this group of patients, we compared it with the CHADS_2_ score and also with two scoring models, which, in addition to the CHA_2_DS_2_-VAS_C_ score components, used the presence of atrial fibrillation and renal failure (shown to be independent predictors of mortality in these patients). The CHA_2_DS_2_-VAS_C_AR score had two additional points for the presence of AF and renal failure (creatinine above 1.1 mg/dL), whereas the CHA_2_DS_2_-VAS_C_AR_2_ score gave an additional point to severe renal failure (creatinine above 2.0 mg/dL). The distribution of mortality according to these scores is shown in Table 7. According to this data, the cutoff values of these scores to discriminate between low and high mortality were 2 for the CHADS_2_ score vs. 4 for the CHA_2_DS_2_-VAS_C_AR and CHA_2_DS_2_-VAS_C_AR_2_ scores (Table 7D).

**Figure 4:**
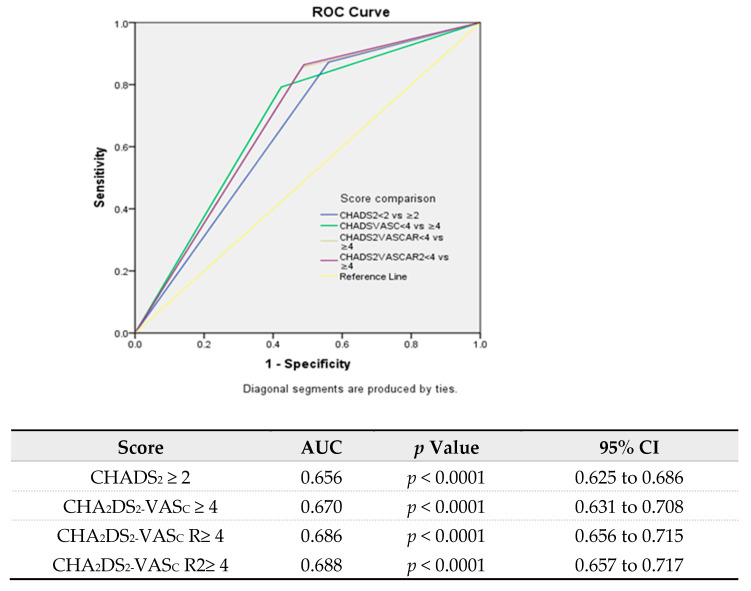

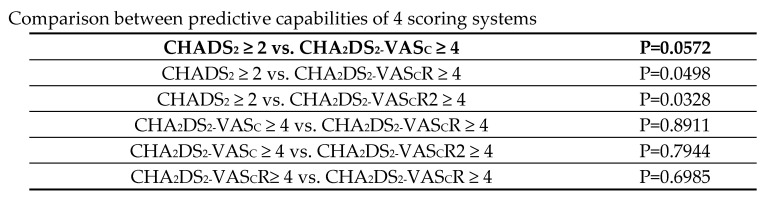
Comparison of the ROC curves of the predictive probability of mortality of the CHA_2_DS_2_, CHA_2_DS_2_-VASC, CHA_2_DS_2_-VASCAR, and CHA_2_DS_2_-VASCAR_2_ scores.

The comparison of the ROC curves using the DeLonge method demonstrated that the CHADS_2_ score performed numerically less well than the other scores, with lower c-statistics almost reaching statistical significance. There was no difference between the CHA_2_DS_2_-VAS_C_, CHA_2_DS_2_-VAS_C_AR, and CHA_2_DS_2_-VAS_C_AR_2_ scores in the predictive capability (Figure 4).

## 4. Discussion

The major findings in our study are:

1. There was an independent association between increased CHA_2_DS_2_-VAS_C_ scores and mortality in patients undergoing coronary angiography.
2. This association was present across multiple subgroups of patients with different clinical characteristics.

Although CHADS_2_ and CHA_2_DS_2_-VAS_C_ scores were initially developed to predict stroke in patients with atrial fibrillation [1,2,3], they were later used to predict multiple cardiovascular outcomes in different categories of patients [7,8,11,12,13,14,18,19,20,21,23,25,26,27,29,30,31,34,35,36,37,38,39,40,41,42].

In our study, a higher (≥4) CHA_2_DS_2_-VAS_C_ score was associated with more significant obstructive coronary artery disease, both in frequency (68.0% vs. 47.6%) and severity (3 vessel CAD present in 44.2% vs. 23.3%). Coronary calcification was also much more frequent in patients with higher scores (31.9% vs. 13.9%). Similar findings were reported by Uysal et al. [29] in STEMI patients and by Cetin et al. [30] in patients who underwent coronary angiography. We used the presence of obstructive CAD and 3-Vessel CAD as markers of significant atherosclerotic coronary disease, because both Gensini and Synthax scores, used by Cetin et al. [30] and Uysal et al. [29], are rarely used in routine clinical practice.

The most important finding of our study was the ability of the CHA_2_DS_2_-VAS_C_ score to predict mortality in a real-life patient population undergoing coronary angiography, as demonstrated by the survival Kaplan–Meier analysis.

Unlike Uysal et al. [29] and Cetin et al. [30], who used modified scores, we used unmodified CHA_2_DS_2_-VAS_C_ scores for our analysis due to its widespread acceptance and convenience. There was a linear association between the CHA_2_DS_2_-VAS_C_ score and mortality from 0 to 6–7 and above. After determining the optimal cutoff value (≥4), its c-statistics (0.670) was better than cited by Puurunen et al. [36] and similar to that of Chan et al. [11]. Additionally, the CHA_2_DS_2_-VAS_C_ score performs similar to other scores, such as Gensini (AOC = 0.63–0.67) [42] and SYNTAX scores (AOC = 0.62–0.67) [43,44], even when used with clinical and biomarker enhancements. It also performed similar to GRACE (AOC = 0.69) [45] and better than TIMI scores (AOC = 0.52) [44]. This was also demonstrated in the study by Huahg et al. [46]. This further validates the use of CHA_2_DS_2_-VAS_C_ scores for mortality prediction.

The multivariate analysis done with the Cox regression model demonstrated that the CHA_2_DS_2_-VAS_C_ score was associated with a two-fold increase in mortality independent from renal function, LVEF, anemia, aortic stenosis, the presence of acute coronary syndrome, obstructive CAD, and atrial fibrillation.

Several studies have previously demonstrated the prognostic value of CHADS_2_ and/or CHA_2_DS_2_-VAS_C_ scores in predicting mortality and MACE in patients with CAD. Most of them [34,35,38,40] studied patients with acute coronary syndrome; however, others included nonurgent patients undergoing coronary angiography or outpatients [11,36,43]. Some studies included only patients with atrial fibrillation [36], some without AF [11], and others demonstrated the values of the CHADS_2_ and CHA_2_DS_2_-VAS_C_ scores in all patients [29,35,38,40]. Crandall et al. [43] and Poçi et al. [34] demonstrated the prognostic value of the CHADS_2_ score separately for both groups of patients (with and without AF). Our Kaplan–Meier analysis demonstrated that the same is true for the CHA_2_DS_2_-VAS_C_ score. However, in our study, beyond demonstrating the usefulness of the CHA_2_DS_2_-VAS_C_ score to predict mortality in both patients with and without atrial fibrillation, the Kaplan–Meier analysis demonstrated the impact of the CHA_2_DS_2_-VAS_C_ score as a predictor of mortality in different categories of patients, i.e., normal vs. reduced LVEF, with ACS vs. elective coronary angiography, with normal or reduced kidney function, and with and without obstructive coronary artery disease. This demonstrated the applicability of the CHA_2_DS_2_-VAS_C_ score in mortality prediction.

We also demonstrated that modification of the CHA_2_DS_2_-VAS_C_ score to include renal failure (CHA_2_DS_2_-VAS_C_R and CHA_2_DS_2_-VAS_C_R_2_) does not provide superior results in terms of the effective discrimination between patients with an increased vs. not increased risk of mortality. Such scores are cumbersome to calculate and not more useful than the familiar CHA_2_DS_2_-VAS_C_ score. This was also similar to the results of the study by study by Huahg et al. [46], where similar R_2_CHA_2_DS_2_-VAS_C_ scores were developed, but the discriminative capability to predict mortality was similar to CHA_2_DS_2_-VAS_C_ scores. It should be noted, however, that the mean CHADSVASC score was much lower in the patients studied by Huang [46] than in our study (2.4 vs. 3.4). Our study demonstrated that the CHA_2_DS_2_-VAS_C_ score is useful in predicting mortality in sicker and older patients.

The multivariate analysis performed in these subgroups demonstrated independent predictions of mortality in all subgroups, apart from patients with preserved LVEF, AF, and the absence of obstructive CAD (even there, a trend was demonstrated, which would probably have reached significance with a larger number of patients).

In summary, our results demonstrate that CHA_2_DS_2_-VAS_C_ scores done before coronary angiography can reliably predict mortality in diverse categories of patients. Although other risk score models (like Gensini and GRACE) have been developed to predict outcomes in patients with MI, the advantage of the CHA_2_DS_2_-VAS_C_ score is its simplicity to calculate. Moreover, the CHA_2_DS_2_-VAS_C_ score is useful not only in AMI but also in elective coronary angiography patients. The CHA_2_DS_2_-VAS_C_ score is an attractive tool for prognosis prediction in patients undergoing coronary angiography due to its simplicity, its good predictive capability, and its applicability in many different subgroups of patients.

### Limitations

First of all, this is a single center observational study with an inherent selection bias. Secondly, only a limited number of patients were involved in this registry. Additionally, the population registry database used to assess survival did not state the diagnosis or whether the death was from cardiac or noncardiac cause.

## 5. Conclusions

The CHA_2_DS_2_-VAS_C_ score can be used as a reliable mortality predictor in patients undergoing coronary angiography. Its prediction is valid in patients with and without atrial fibrillation, preserved and reduced left ventricular function, with and without renal failure, and in both elective and urgent angiography.

## Figures and Tables

**Table 1:** Distribution of patients according to their CHA_2_DS_2_-VAS_C_ scores.

CHA_2_DS_2_-VAS_C_ Score	# of Patients	Percent	Mortality (%)
0	37	3.7	5.4
1	115	11.6	7.8
2	176	17.8	13.6
3	199	20.1	16.6
4	206	20.8	33.5
5	150	15.2	42.0
6	78	7.9	44.9
7+	29	2.9	44.8
Total	990	100	25.1

*p*-value < 0.001.

**Table 2:** Effect of individual components of the CHA_2_DS_2_-VAS_C_ score on mortality.

Variable	Mortality If Absent (%)	Mortality If Present (%)	*p*-Value
Age ≥ 65	4.3	17.9	<0.0001
Age ≥ 75	8.0	23.1	<0.0001
Female Gender	11.2	16.8	0.011
HTN	5.9	15.3	<0.0001
DM	9.8	17.0	<0.0001
Vascular Disease	9.7	15.0	0.009
CHF	10.0	29.9	<0.0001
Previous stroke	12.3	18.1	0.082

Abbreviations: HTN: hypertension, DM: diabetes mellitus, and CHF: chronic heart failure.

**Table 3:** Baseline patient characteristics according to the CHA_2_DS_2_VAS_C_ scores.

Variable	Total Patients 990 (100%)	Patients with CHA_2_DS_2_-VAS_C_ < 4 527 (53.2%)	Patients with CHA_2_DS_2_-VAS_C_ ≥ 4 463 (46.8%)	*p*-Value
Age	68.1 ± 11.7	61.7 ± 10.7	75.43 ± 8.71	<0.0001
Female	30.3	18.0	44.3	<0.0001
AF	19.6	12.4	27.8	<0.0001
DM	43.6	25.9	63.7	<0.0001
HTN	75.8	59.0	94.8	<0.0001
Dyslipidemia	70.6	65.1	76.9	<0.0001
Previous MI	22.3	18.5	26.5	0.003
Previous PCI	33.5	29.7	37.9	0.007
Previous CABG	22.8	15.4	31.0	<0.0001
Previous CVA	9.7	0.4	20.1	<0.0001
PVD	6.1	3.7	8.7	0.001
CHF	14.3	8.2	21.2	<0.0001
GFR < 60 mL/min/1.73 m^2^	43.2	21.1	68.0	<0.0001
ACS	49.1	48.5	49.7	0.727
Obstructive CAD	57.2	47.6	68.0	<0.0001
3 Vessel CAD	33.0	23.3	44.2	<0.0001
Calcified plaques	22.2	13.9	31.9	<0.0001
Elevated LV filling pressure	32.3	21.9	42.8	<0.0001
EF > 50%	48.2	49.5	46.8	0.461
Anemia (HB < 13 g/dL)	43.1	27.4	60.7	<0.0001
AS	19.5	11.9	28.0	<0.001
BMI	28.7 ± 5.2	28.7 ± 5.1	28.7 ± 5.23	0.869
EF	49.3 ± 9.9	49.8 ± 9.4	48.8 ± 10.4	0.170
Mortality	25.1	13.1	38.7	<0.0001

Continuous values are presented as the mean ± standard deviation. Dichotomic variables are presented as the percentage of the total number of patients in the relevant rubric. Abbreviations: AF: atrial fibrillation, HTN: hypertension, DM: diabetes mellitus, MI: myocardial infarction, PCI: percutaneous coronary intervention, CABG: coronary artery bypass graft, CVA: cerebrovascular accident, PVD: peripheral vascular disease, CHF: chronic heart failure, GFR: glomerular filtration rate, ACS: acute coronary syndrome, CAD: coronary artery disease, LV: left ventricular, EF: ejection fraction, AS: aortic stenosis, and BMI: body mass index.

**Table 4:** Univariate analysis of the association of different variables with mortality.

Variable	Total Patients 990 (%)	Alive 742 (74.9%)	Deceased 249 (25.1%)	*p* Value
Age	68.1 ± 11.7	65.9 ± 11.5	74.9 ± 9.7	<0.0001
Female	30.3	29.4	33.1	0.274
AF	19.6	15.5	31.9	<0.0001
DM	43.6	40.9	51.6	0.004
HTN	75.8	73.0	84.3	<0.0001
Dyslipidemia	70.6	70.4	71.4	0.750
Previous MI	22.3	22.5	21.6	0.779
Previous PCI	33.5	32.7	36.2	0.311
Previous CABG	22.8	21.0	28.0	0.023
Previous CVA	9.7	8.8	12.2	0.08
PVD	6.1	3.6	13.4	<0.0001
CHF	14.3	9.5	28.5	<0.0001
GFR < 60 mL/min/1.73 m^2^	43.2	33.3	72.8	<0.0001
ACS	49.1	47.9	52.5	0.125
Obstructive CAD	57.2	56.2	60.1	0.285
3 Vessel CAD	33.0	30.8	39.6	0.015
Calcified	22.2	19.3	30.8	0.001
Elevated LV filling pressure	32.3	22.6	54.8	<0.0001
EF > 50	48.2	52.3	37.6	<0.0001
Anemia (HB < 13 g/dL)	43.1	36.1	63.1	<0.0001
AS	19.5	13.9	33.8	<0.0001
CHA_2_DS_2_VAS_C_ ≥ 4	46.8	38.3	72.2	<0.0001
BMI	28.7 ± 5.2	28.8 ± 5.0	28.3 ± 5.7	0.191
EF	49.3 ± 9.9	50.5 ± 9.0	46.1 ± 11.3	<0.0001
CHADS_2_ Score	1.84 ± 1.22	1.65 ± 1.19	2.44 ± 1.13	<0.0001
CHA_2_DS_2_-VAS_C_ Score	3.35 ± 1.71	3.05 ± 1.67	4.24 ± 1.53	<0.0001

Continuous values are presented as the mean ± standard deviation. Dichotomic variables are presented as the percentage of the total number of patients in the relevant rubric. Abbreviations: AF: atrial fibrillation, HTN: hypertension, DM: diabetes mellitus, MI: myocardial infarction, PCI: percutaneous coronary intervention, CABG: coronary artery bypass graft, CVA: cerebrovascular accident, PVD: peripheral vascular disease, CHF: chronic heart failure, GFR: glomerular filtration rate, ACS: acute coronary syndrome, CAD: coronary artery disease, LV: left ventricular, EF: ejection fraction, AS: aortic stenosis, and BMI: body mass index.

**Table 5:** Cox regression multivariate analysis of mortality.

Variable	Hazard Ratio	CI	*p*-Value
Atrial fibrillation	1.23	0.85–1.76	0.272
CHA_2_DS_2_-VAS_C_ ≥ 4	2.14	1.40–3.26	<0.0001
GFR < 60 mL/min/1.73 m^2^	2.16	1.40–3.22	0.001
Ejection fraction < 50%	1.64	1.17–2.28	0.004
Anemia	1.51	1.06–2.13	0.021
Elevated LV filling pressure	1.95	1.40–2.73	<0.0001
Acute coronary syndrome	1.08	0.78–1.49	0.655
Obstructive CAD	1.07	0.76–1.50	0.715
Aortic Stenosis	1.40	0.98–2.00	0.067

Abbreviations: CAD: coronary artery disease.

**Table 6:** Cox regression multivariate analysis of mortality: CHA_2_DS_2_-VAS_C_ score (<4 vs. ≥4) significance in the different prespecified subgroups.

Subgroup	Hazard Ratio	CI	*p*-Value
Non ACS	2.11	1.13–3.95	0.019
ACS	2.178	1.22–3.89	0.009
No obstructive CAD	1.74	0.95–3.19	0.074
Obstructive CAD	2.80	1.52–5.16	0.001
Reduced EF	2.63	1.56–4.41	<0.0001
Preserved EF	1.632	0.78–3.37	0.197
Normal Kidney Function	2.54	1.29–5.00	0.007
Reduced Kidney Function	1.878	1.11–3.18	0.019
No AF	2.36	1.43–3.91	0.001
AF	1.94	0.97–3.86	0.06

Abbreviations: AF: atrial fibrillation, ACS: acute coronary syndrome, CAD: coronary artery disease, and EF: ejection fraction.

**Table 7:** Distribution of mortality according to the CHADS_2_, CHA_2_DS_2_-VAS_C_AR, and CHA_2_DS_2_-VAS_C_ AR_2_ scores.

CHADS_2_ Score				
CHADS_2_ Score	Frequency	Percent	Cumulative Percent	Mortality (%)
0	142	14.4	14.4	4.2
1	256	26.0	40.4	4.3
2	318	32.3	72.6	12.6
3	188	19.1	91.7	25
4	54	5.5	97.2	27.8
5	28	2.8	100.0	32.1
Total	986	100.0	100.0	13
CHA_2_DS_2_-VAS_C_**AR score**				
0	32	3.3	3.3	0
1	102	10.6	13.9	4.9
2	135	14.0	27.9	3.0
3	182	18.9	46.8	4.9
4	154	16.0	62.8	9.1
5	160	16.6	79.4	18.1
6	115	11.9	91.3	24.3
7	57	5.9	97.2	38.6
8	21	2.2	99.4	47.6
9	6	0.6	100.0	66.7
Total	964	100.0	100.0	
CHA_2_DS_2_-VAS_C_**AR_2_ score**				
0	32	3.3	3.3	0
1	101	10.5	13.8	5.0
2	135	14.0	27.8	3.0
3	178	18.5	46.3	4.5
4	157	16.3	62.6	8.3
5	147	15.2	77.8	18.4
6	123	12.8	90.6	22.8
7	55	5.7	96.3	41.8
8	28	2.9	99.2	39.3
9	7	0.7	99.9	71.4
10	1	0.1	100.0	100
Total	964	100.0	100.0	
Comparison between low and high mortality groups according to the aforementioned scores				
			Mortality	
CHADS_2_ < 2			4.3%	
CHADS_2_ ≥ 2			18.9%	
CHA_2_DS_2_-VAS_C_ < 4			5.3%	
CHA_2_DS_2_-VAS_C_ ≥ 4			21.7%	
CHA_2_DS_2_-VAS_C_AR < 4			4.0%	
CHA_2_DS_2_-VAS_C_AR ≥ 4			20.9%	
CHA_2_DS_2_-VAS_C_AR_2_ < 4			3.8%	
CHA_2_DS_2_-VAS_C_AR_2_ ≥ 4			20.9%	

*p* < 0.0001 for any difference in mortality. *p* < 0.0001 for all.

## Data Availability

The data were securely preserved on a dedicated server at the Kaplan Medical Center. According to the Clalit HMO policy, the release of data to a third party is restricted. Denominated data might be provided to a third party (i.e., editors and reviewers) after obtaining permission from the legal department of the medical center.

## Abbreviations

ACS: acute coronary syndrome; AF: atrial fibrillation; CHF: chronic heart failure; CKD: chronic kidney disease; LVEF: Left ventricular ejection fraction; PVD: peripheral vascular disease; GFR: glomerular filtration rate; CAD: coronary artery disease.
